# Thy-1 predicts poor prognosis and is associated with self-renewal in ovarian cancer

**DOI:** 10.1186/s13048-019-0590-5

**Published:** 2019-11-17

**Authors:** Elizabeth V. Connor, Caner Saygin, Chad Braley, Andrew C. Wiechert, Sheelarani Karunanithi, Katie Crean-Tate, Fadi W. Abdul-Karim, Chad M. Michener, Peter G. Rose, Justin D. Lathia, Ofer Reizes

**Affiliations:** 1 Division of Gynecologic Oncology, Department of Obstetrics and Gynecology and Women’s Health Institute, Cleveland Clinic, 801 North 29th Street Billings, MT, Cleveland, OH 59101 USA; 2 Billings Clinic Cancer Center, Division of Gynecologic Oncology, 801 North 29th Street, Billings, MT 59101 USA; 3 Cancer Impact Area, Lerner Research Institute, Cleveland Clinic, Cleveland, OH USA; 4 Center of Excellence in Gynecological Cancer Research, Cleveland Clinic, Cleveland, OH USA; 5 Department of Anatomic Pathology, Cleveland Clinic, Cleveland, OH USA; 6 Cleveland Clinic Lerner College of Medicine of Case Western Reserve University, Cleveland, OH USA; 7 Cardiovascular and Metabolic Sciences, Lerner Research Institute, Cleveland Clinic Foundation, 9500 Euclid Avenue, Desk NC10, Cleveland, OH 44195 USA; 8 Case Comprehensive Cancer Center, Cleveland, OH USA

**Keywords:** Ovarian cancer, Thy-1, CD90, Cancer stem cells, Biomarker, Self-renewal

## Abstract

**Background:**

Ovarian cancer is the leading cause of gynecologic cancer death in the United States despite effective first-line systemic chemotherapy. Cancer stem cells (CSCs) retain the ability to self-renew and proliferate and may be a means of harboring disease that evades standard treatment strategies. We previously performed a high-throughput screen to assess differential protein expression in ovarian CSCs compared to non-CSCs and observed that Thy-1 was more highly expressed in CSCs. Our primary aim was to validate Thy-1 (CD90) as a cancer stem cell (CSC) marker in epithelial ovarian cancer (EOC), correlate with clinical outcomes, and assess as a potential therapeutic target.

**Results:**

Kaplan Meier (KM) Plotter data were correlated with survival outcomes. Quantitative real-time PCR, flow cytometry, and immunoblots assessed RNA and protein expression. Limiting dilution assays assessed self-renewal capacity and proliferation assays assessed proliferative capacity. RNA in-situ hybridization was performed on patient specimens to assess feasibility. Thy-1 (CD90) is more highly expressed in ovarian CSCs than non-CSCs, in EOC compared to benign ovarian epithelium (*P* < 0.001), and is highest in serous EOC (*P* < 0.05). Serous ovarian cancers with high Thy-1 expression have poorer outcomes (median PFS 15.8 vs. 18.3 months, *P* = 0 < 0.001; median OS 40.1 v. 45.8 months, *P* = 0.036). Endometrioid ovarian cancers with high Thy-1 have poorer PFS, but no difference in OS (upper quartile PFS 34 v. 11 months, *P* = 0.013; quartile OS not reached, *P* = 0.69). In vitro, Thy-1 expression is higher in CSCs versus non-CSCs. EOC cells with high Thy-1 expression demonstrate increased proliferation and self-renewal. Thy-1 knockdown in EOC cells decreases proliferative capacity and self-renewal capacity, and knockdown is associated with decreased expression of stem cell transcription factors NANOG and SOX2. RNA in situ hybridization is feasible in ovarian cancer tissue specimens.

**Conclusions:**

Thy-1 is a marker of ovarian CSCs. Increased expression of Thy-1 in EOC predicts poor prognosis and is associated with increased proliferative and self-renewal capacity. Thy-1 knockdown decreases proliferative and self-renewal capacity, and represents a potential therapeutic target.

## Background

Ovarian cancer is the leading cause of gynecologic cancer-related death in the United States [1]. Currently there are no effective screening modalities available and over 80% of patients present with advanced stage disease [2]. Even with aggressive cytoreductive surgery and adjuvant chemotherapy, over 80% of patients with advanced stage disease will recur [3]. Furthermore, recurrent disease often demonstrates increasing resistance to conventional chemotherapy and contributes to the high mortality of this disease [4].

Cancer stem cells (CSCs) are cancer cells that retain the ability to self-renew and exhibit increased proliferation and chemoresistance [5]. In ovarian cancer, CSCs have been suggested as a means of chemoresistance and aggressive malignant behavior and thus are attractive therapeutic targets [6]. Successful identification of CSCs in ovarian cancer may be helpful in determining and subsequently targeting mechanisms of chemoresistance and recurrence in the future. Several markers have been implicated in ovarian cancer, including CD133, CD44, CD24, CD117, EpCAM and ALDH [7]. We previously transduced ovarian cancer cells (A2780) with a NANOG-GFP reporter system to identify ovarian CSCs based on GFP intensity [8, 9]. Using this platform, we performed a high-throughput flow cytometry screen to compare expression of 242 cell surface markers in ovarian CSCs (GFP-positive) and non-CSCs (GFP-negative) and identified CD55 as a CSC marker and a driver of self-renewal and chemoresistance pathways [10–12]. Our high-throughput screen also identified a second protein that was more highly expressed in CSCs, Thy-1.

Thy-1 (also known as CD90) is a glycosylphosphatidylinositol (GPI) anchored protein that localizes to lipid rafts at the cell surface [13, 14]. Investigation of the role of Thy-1 in ovarian cancer is limited. Abeysingh et al. investigated the effect of Thy-1 overexpression on tumorigenicity of the SKOV3 established cell line and suggested that Thy-1 regulates differentiation and acts as a putative tumor suppressor [15, 16]. This is in stark contrast to more recent discovery that Thy-1 is a CSC marker in glioblastoma as well as hepatocellular, pancreatic, and gallbladder cancer and promotes tumorigenicity and self-renewal [17–21]. Our high-throughput screen suggested Thy-1 as a putative CSC marker in ovarian cancer. Our primary objective was to validate this finding with in vitro studies and to correlate with clinical outcomes in women with ovarian cancer. Our secondary objective was to evaluate whether we could target Thy-1 to negatively impact cancer cell growth.

## Results

### Thy-1 is more highly expressed in ovarian CSCs compared to non-CSCs

**Fig. 1:**
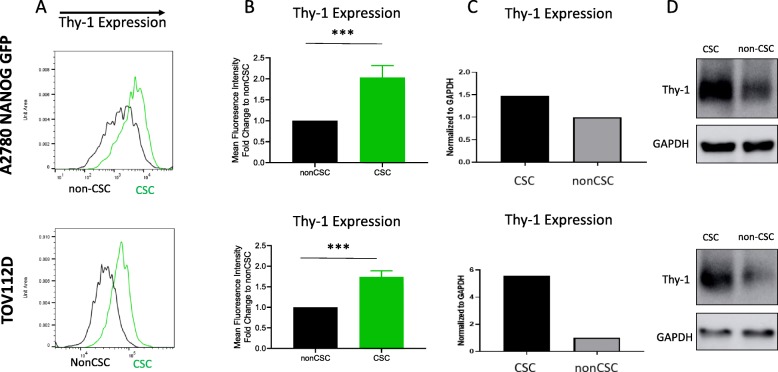
Thy-1 is highly expressed in ovarian cancer stem cells. A high throughput screen identified Thy-1 as one of two cell surface proteins more highly expressed in NANOG-GFP+ A2780 ovarian CSCs compared to non-CSCs and this was also observed in CD49f + TOV112D ovarian cancer CSCs (**a, b**). We subsequently validated in RNA via qRT-PCR (**c**) and protein via Western Blot (**d**)

**Fig. 2:**
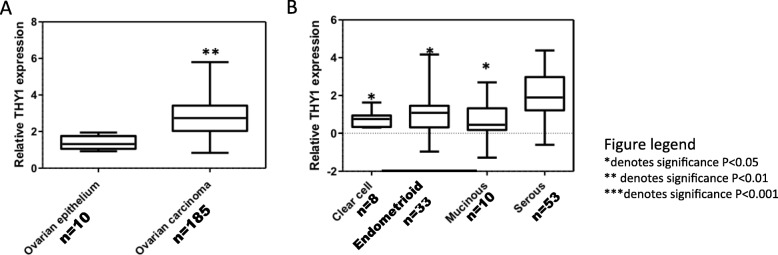
Thy-1 is highly expressed in ovarian cancer cells, especially serous adenocarcinoma**.** Bioinformatic analysis of ovarian tissue databases demonstrated higher expression of Thy-1 in cancerous versus benign ovarian tissue epithelium (**a**) and highest expression in serous and endometrioid histologic subtypes (**b**)

We previously described and validated a NANOG promotor-driven GFP reporter system for isolating ovarian CSCs (GFP-positive) from non-CSCs (GFP-negative) and subsequently reported our finding that CD55, a cell surface complement inhibitor, was differentially expressed in ovarian CSCs as compared to non-CSCs [9, 12]. This led to the discovery that CD55 regulates self-renewal and cisplatin resistance via a bifurcating signaling axis to promote cell survival and chemoresistance [12]. From a total of 242 cell surface proteins that were included in this high-throughput screen, we identified Thy-1 was also more highly expressed in ovarian CSCs as compared to non-CSCs in A2780 cisplatin-naïve ovarian cancer cells (Fig. 1a, b). We then demonstrated 12-fold higher expression of Thy-1 at the RNA level in GFP+ A2780 ovarian cancer cells compared to GFP- A2780 cells (Fig. 1c). Protein expression findings from the high-throughput screen were also confirmed on Western blot (Fig. 1d). We next sought to examine the clinical relevance of this finding by examining bioinformatic data in the publicly accessible Bonome and Schwartz ovarian datasets [22, 23]. We identified significantly higher expression of Thy-1 in ovarian carcinoma compared to benign ovarian epithelium (Fig. 2a). Using available data from the Schwartz ovarian dataset, we identified highest expression of Thy-1 in serous and endometrioid histologic subtypes of ovarian cancer (Fig. 2b).

### Thy-1 expression is associated with poorer clinical outcomes

**Fig. 3:**
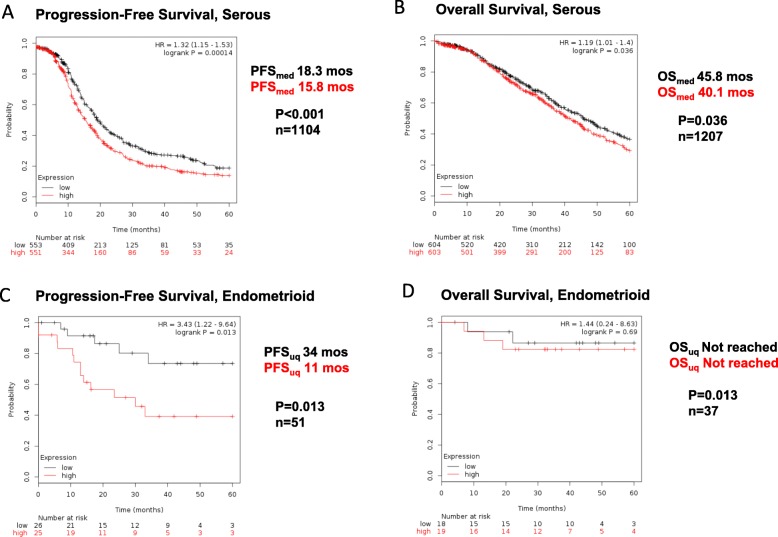
Thy-1 is associated with poorer clinical outcomes in women with ovarian cancer. High expression of Thy-1 is associated with poorer progression-free and overall survival in serous ovarian cancer (**a, b**), and poorer progression-free survival, but not overall survival in endometrioid ovarian cancer (**c, d**)

Since we had noted that Thy-1 expression was higher in ovarian CSCs, we hypothesized that Thy-1 may act as a surrogate marker for stem cell populations in women with ovarian cancer and therefore may be a relevant indicator of prognosis. We used Kaplan-Meier survival analysis to investigate clinical outcomes in women with serous and endometrioid ovarian cancer. In women with serous ovarian cancer, Thy-1 expression was associated with a significantly poorer median progression-free survival (15.8 vs. 18.3 months, *P* < 0.001, *n* = 1104, Fig. 3a), and significantly poorer median overall survival (40.1 vs 45.8 months, *P* = 0.036, *n* = 1207, Fig. 3b). Of note, for women with endometrioid ovarian cancer, median PFS comparison was not calculable as there were far few recurrence and death events in this group. Accordingly, upper quartile was used to better describe differences between the groups. In women with endometrioid ovarian cancer, Thy-1 expression was associated with significantly poorer progression-free survival (upper quartile PFS 11 vs. 34 months, *P* = 0.013, *n* = 51, Fig. 3c). Overall survival was excellent in women with endometrioid ovarian cancers and at a clinical follow-up time of 5 years, neither cohort had reached > 25% mortality events, and there were no significant differences in overall survival by Thy-1 expression (Fig. 3d).

### Thy-1 is associated with increased proliferative and self-renewal capacity

**Fig. 4:**
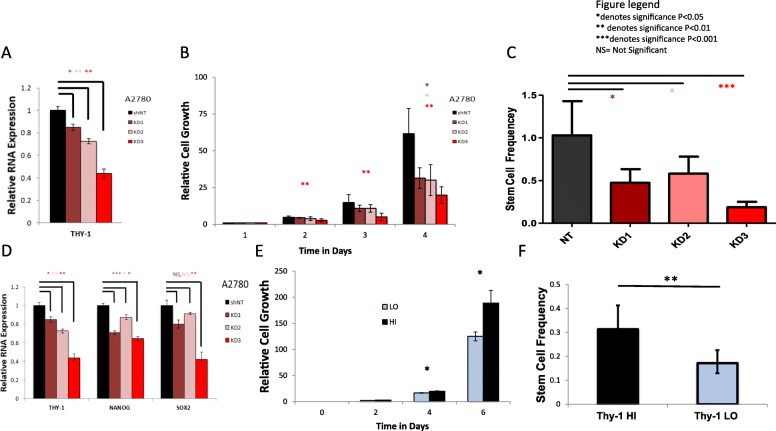
Thy-1 is associated with increased proliferation and self-renewal. Thy-1 knockdowns were generated in A2780 ovarian cancer cells (**a**). Thy-1 knockdown correlated with decreasing proliferation capacity under the same conditions in vitro (**b**), and decreasing self-renewal capacity in a limiting dilution tumor sphere formation assay (**c**). Knockdowns demonstrated lower expression of stem cell transcription factors NANOG and SOX2 (**d**). Subsequently, A2780 ovarian cancer cells were sorted via FACS into Thy-1 enriched (Thy-1 HI) and non-enriched (Thy-1 LO) populations and the Thy-1 enriched population demonstrated increased proliferation (**e**) and self-renewal capacity (**f**). Significance: * *P* < 0.05, ** *P* < 0.01, *** *P* < 0.0001, NS = Not Significant

To further examine the relationship between Thy-1 expression and stemness, we generated and validated Thy-1 knockdowns (Fig. 4, Additional file 2: Figure S2). Next, we performed in vitro cellular proliferation assays to examine differences in proliferation potential in Thy-1 non-targeting controls compared to the Thy-1 knockdowns. We found that Thy-1 knockdowns exhibited decreased proliferation compared to the non-targeting control, and that the level of Thy-1 expression was directly correlated with proliferative capacity (Fig. 4b). We also evaluated self-renewal capacity of Thy-1 knockdowns as compared to non-targeting control using a limiting dilution sphere-forming assay and observed that stem cell frequency was significantly decreased in knockdowns (Fig. 4c). Additionally, we found that knockdowns had lower expression of stem cell transcription factors NANOG and SOX2 (Fig. 4d). After demonstrating loss of proliferative ability and self-renewal in Thy-1 knockdowns, we sought to evaluate whether cell populations with higher Thy-1 expression would demonstrate greater proliferative potential and self-renewal than similar cells with low Thy-1 expression. A2780 ovarian cancer cells were sorted using Fluorescence Activated Cell Sorting (FACS) and Thy-1 high cells demonstrated both higher proliferative capability as well as higher self-renewal on limiting dilution assay (Fig. 4e, f).

### Thy-1 RNA expression is variable in human ovarian tissue

**Fig. 5:**
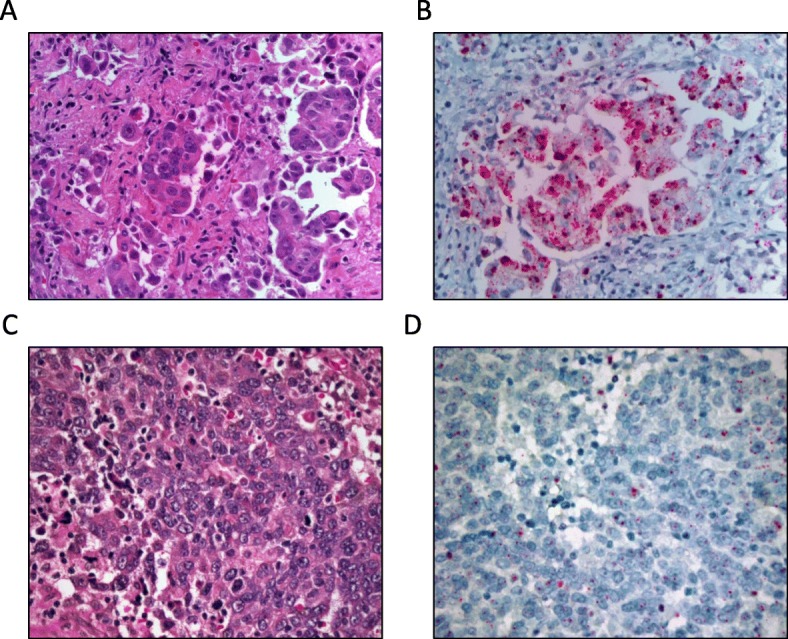
RNA in situ hybridization of human ovarian cancer tissue. RNA in situ hybridization was performed on human ovarian tissue and graded by a pathologist from 1+ to 3+. Both Patient 4 (**a, b**) and Patient 2 (**c, d**) had serous ovarian cancer demonstrated Hematoxylin and Eosin (H&E) at 400x (**a, c**). Patient 4 demonstrated 3+ ISH for Thy-1 (**b**) and Patient 2 demonstrated 1+ ISH for Thy-1 (**d**)

After demonstrating that higher expression of Thy-1 is associated with increased proliferative ability and self-renewal in vitro, we sought to examine whether we could identify Thy-1 expression through RNA in-situ hybridization in human tissue, and whether we could identify variable expression. We previously had examined KM Plotter data that demonstrated poorer progression-free and overall survival in women with serous ovarian cancer and high Thy-1 RNA expression (Fig. 3a, b). In a sample of 7 women with advanced (stage IIIc) high grade serous ovarian cancers and a minimum of 3 years of follow-up data, we identified differential Thy-1 RNA expression (Fig. 5). Clinical outcomes data were collected retrospectively and are presented in Table 1.

**Table 1:**
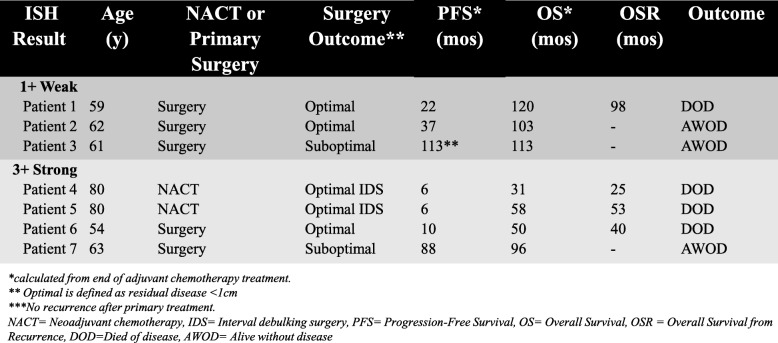
Clinical outcomes of patients selected for RNA in situ hybridization for Thy-1. Table includes clinical outcomes data for patients with tissue analyzed by RNA ISH. PFS and OS are calculated from the completion date of adjuvant chemotherapy. Optimal surgery outcome is defined as residual disease < 1 cm. *** denotes that patient had no recurrence of disease

## Discussion

While a majority of ovarian cancers demonstrate sensitivity to standard chemotherapy, recurrent disease is pervasive, which is why women who present with advanced disease (stage III or IV) currently face survival rates between 19 to 47% at 5 years [24]. The recurrence pattern and eventual development of chemoresistance in ovarian cancer make it an apt target for study of cancer stem cells (CSCs). While several CSC markers have been identified in ovarian cancer cells, none are specific or independently sufficient to consistently identify ovarian CSCs and more investigation is warranted to better characterize ovarian CSCs [25]. We identified Thy-1 as a cell surface marker more highly expressed in a GFP-labeled CSC population and were able to subsequently demonstrate increased proliferation and self-renewal capacity that was reversed by Thy-1 knockdown. Thy-1 may contribute to better identification of the ovarian CSC signature and may help guide efforts in the identification and eventual targeting of CSCs.

Ovarian CSC identification may not only have prognostic implications, but has the potential to guide management decisions. Meng et al. previously found that women with serous ovarian cancer and ascites specimens with higher expression of the CSC marker CD44 had significantly poorer progression-free survival (6 vs. 18 mos, *P* = 0.01) [26]. This is a clinically significant finding, and if validated on a larger scale could help to guide decision-making regarding treatment goals and the role of maintenance therapy. In our bioinformatics analysis, we found that high Thy-1 expression was associated with poorer progression-free survival in both serous and endometrioid ovarian cancers. We identified a particularly compelling difference in progression-free survival for women with endometrioid ovarian tumors with high expression of Thy-1 (Fig. 3c). In women with endometrioid ovarian tumors, women with high expression of Thy-1 were more than 3 times more likely to experience recurrent disease than women with low expression of Thy-1. This trend was consistent in both early stage and late state endometrioid ovarian cancers, although due to limited numbers of endometrioid ovarian tumors, power was limited (Additional file 1: Figure S1). This association is especially relevant in the case of endometrioid tumors because these are frequently well-differentiated (low grade), often confined to the ovary at diagnosis (stage 1a or 1b), and when early stage are associated with a 5-year overall survival of over 90% [27]. Given this, low grade and early stage tumors may be spared adjuvant treatment with chemotherapy and younger patients are sometimes offered fertility-sparing surgery to balance increased surgical morbidity against recurrence risk. In this setting, knowledge of increased CSC expression and corresponding increased recurrence risk may be an indication for closer surveillance or more aggressive clinical management.

To evaluate levels of RNA expression for Thy-1 in human tissue, we selected a small cohort of 7 women with stage IIIc high grade serous ovarian cancer that had undergone surgery and received adjuvant therapy with surveillance at our institution. This was intended as a pilot study and we deliberately selected patients with variable progression-free intervals following platinum-based chemotherapy. We noted incidentally that women with weak Thy-1 staining experienced longer than expected progression-free survival (Table 1). However, the limited number of patients precludes meaningful clinical outcome comparisons. Also, our clinical database included patients undergoing surgery in 2006–2007 and care patterns were different during this time period. For example, of these 7 patients, Patient 6 was known to be BRCA2+ and Patient 1 tested negative for BRCA, however all others had unknown BRCA status. BRCA status is known to be associated with platinum sensitivity and longer progression-free intervals. Additionally, Patients 1 and 5 received adjuvant IP therapy, and both Patients 2 and 6 were enrolled in GOG 218, a blinded study that randomized patients to receive adjuvant carboplatin and paclitaxel with bevacizumab or placebo [28]. Further appropriately powered studies are needed to demonstrate whether RNA in situ hybridization carries prognostic value for ovarian cancer; however, our data do support that expression is differential.

Beyond prognostic value, CSC markers are potential targets for preventing recurrence and neutralizing chemoresistance. After treatment with neoadjuvant chemotherapy, patient tissue samples of residual ovarian cancer have been shown to display enriched stem cell populations [29]. Interestingly, both patients in our small cohort that were treated with neoadjuvant chemotherapy had strong RNA expression of Thy-1, which raises the question of whether neoadjuvant chemotherapy enriches Thy-1 expressing cell populations. CSCs are known to have multiple features that are thought to contribute to chemoresistance including more efficient DNA protection and repair mechanisms, activation of survival pathways, and inactivation of apoptotic pathways [25]. Chau et al. previously identified a population of CD117+ (c-kit) ovarian cancer cells and demonstrated reduced resistance to cisplatin and paclitaxel in a murine model with c-kit knockdown and treatment with imatinib, a tyrosine kinase inhibitor known to target c-kit [30]. Zhang et al. previously used miRNA to target Thy-1+ CSCs in a murine model of hepatocellular carcinoma [31]. We were able to demonstrate decreased proliferation and self-renewal after Thy-1 knockdown in ovarian cancer cells (Fig. 4b, c), suggesting a potential for targeting. The ability to specifically target CSCs in ovarian cancer would hopefully allow oncologists to reduce recurrence risk by targeting those cells that are able to evade conventional systemic chemotherapy and retain the ability to grow and metastasize.

Despite promising results in vitro and in vivo models, early translational studies in ovarian cancer have been disappointing. However, the relapsing nature of ovarian cancer begs the question whether targeted therapy is being appropriately investigated. For example, early clinical trials of imatinib (which targets c-kit, CD117) in women with ovarian cancer have failed to show a survival benefit, even when evaluated in women with increased expression of associated biomarkers [32–35]. However, each of those studies evaluated efficacy of an oral drug in women with platinum-resistant, active recurrent metastatic disease. If CSCs are promoters of cell population survival and recurrence, perhaps these targeted agents are best studied as maintenance therapies for women with minimal to no visible residual disease. Or, perhaps CSC-targeted therapy should be studied in the same spaces in which microscopic residual disease exists, such as the peritoneal cavity. Both intraperitoneal delivery of chemotherapy and intraoperative delivery of heated intraperitoneal chemotherapy have been shown in prospective randomized trials to prolong the disease-free interval in ovarian cancer, but the impact that these treatment modalities have on CSCs is unknown [36, 37]. It would be valuable in future prospective studies to collect and analyze tissue with the goal of understanding how treatment impacts CSC populations and whether this correlates with survival outcomes.

## Conclusions

In summary, we identified Thy-1 as a surface marker of CSCs in ovarian cancer and further demonstrated increased proliferative and self-renewal capability in Thy-1 expressing cells that was reversible with Thy-1 knockdown (Figs. 1,4). We were additionally able to validate these findings by identifying poorer clinical outcomes for women with ovarian cancer and high expression of Thy-1 (Fig. 3). The paradox of ovarian cancer is that although we have generally efficacious chemotherapy agents in the primary setting, we fail to target microscopic cells that escape primary therapy and progress into recurrent, almost universally fatal, disease. It is imperative that we further investigate CSC markers in ovarian cancer and understand the role of CSCs as predictors and mediators of recurrence so that we can target these cells with the goal of keeping patients disease-free.

## Methods

### Cell culture

The ovarian cancer cell line A2780 (cisplatin naive) was cultured in DMEM supplemented with 10% fetal bovine serum (FBS) at 37 °C in a humidified atmosphere (5% CO_2_). The ovarian cancer cell line TOV211D was cultured in MCDB 105 medium supplemented with 15% FBS at 37 °C in a humidified atmosphere (5% CO_2_). Cell lines were acquired from the American Type Culture Collection (ATCC) and underwent short tandem repeat (STR) DNA profiling analysis. At 70–90% confluence, trypsin/EDTA was applied to cell culture to extract cells for use in experiments for further passaging.

### Flow cytometry and high-throughput screen

Our lab previously developed NANOG-GFP promoter-transduced A2780 ovarian cancer cells to allow for reliable sorting of stem and non-stem population of ovarian cancer cells [9]. NANOG-GFP transduced A2780 ovarian cancer cells were prepared to a concentration of 1 million cells/mL and were sorted using the BD FACS Aria II platform to isolate GFP-high and GFP-low cell populations. GFP-high cell populations are considered cancer stem cells (CSCs), and GFP-low cell populations are considered non-cancer stem cells (non-CSCs). APC-conjugated Thy-1(1:100, BD Biosciences) was used for FACS analysis and gates were set to the top and bottom 10% of expression to represent Thy-1 high and low populations respectively.

High-throughput flow cytology screening was performed as previously reported using the BD Lyoplate Human Cell Surface Marker Screening Panel (BD Biosciences) which is a monoclonal antibody panel that includes 242 cell surface markers as well as mouse and rat controls to account for background signal [9]. Plates were analyzed on a Fortessa HTS system (BD Biosciences) and all data were analyzed with FlowJo software (Tree Star).

### Lentivirus production and transfection for generation of knockdowns

Lentiviral shRNAs were developed for Thy-1 as previously reported [11, 38]. HEK 293 T cells were co-transfected with the packaging vectors pMD2.G and psPAX2 (Addgene) and lentiviral vectors for expression of shRNA specific to Thy-1 (Sigma-Aldrich) and a nontargeting control shRNA were applied. Media were changed 24 h after transfection, and at 48 h viral material was harvested using polyethylene glycol precipitation and stored at − 80 °C. A2780 cell lines were infected with viral material and after transduction underwent puromycin selection.

### Quantitative real time PCR (qRT-PCR)

RNA was extracted from A2780 control and Thy-1 knockdowns using RNeasy kit (Qiagen). cDNA was made using 1 μg of total RNA using the Superscript III kit (Invitrogen, Grand Island, NY). SYBR Green-based real time PCR was performed using SYBR-Green master mix (BA Biosciences) and the Applied Biosystems StepOnePlus real time PCR machine (Thermo). Each experiment was run in triplicate. The threshold cycle (Ct) values for each gene were normalized to β-actin. PCR primer sequences used included:

β-actin Forward 5′-AGAAAATCTGGCACCACACC-3′.

Reverse 5′-AGAGGCGTACAGGGATAGCA-3′.

Thy-1 Forward 5′- GAGATCCCAGAACCATGAACC − 3′.

Reverse 5′- TGCTGGTATTCTCATGGCG -3′.

NANOG Forward 5′-CCCAAAGGCAAACAACCCACTTCT-3′.

Reverse 5′-AGCTGGGTGGAAGAGAACACAGTT-3′.

SOX2 Forward 5′-CACATGAAGGAGCACCCGGATTAT-3′.

Reverse 5′-GTTCATGTGCGCGTAACTGTCCAT-3′.

### Western blotting

To create cell protein extracts, cells were lysed in 20 mM Tris-HCl (pH 7.5), 150 mM NaCl, 1 mM Na_2_EDTA, 1% NP-40, 1 mM EGTA, 1% sodium pyrophosphate, 1 mM β-glycerophosphate, 1 mM sodium orthovanadate, 1 μg/mL leupeptin, 20 mM NaF and 1 mM PMSF. Protein concentrations were determined using a standard Bradford reagent (BIO-RAD). Protein lysates (30–50 μg of total protein) were resolved by 10% SDS-PAGE and transferred to nitrocellulose membrane. Membranes were incubated overnight at 4 °C with primary antibodies against Thy-1(1:1000) (Cell Signaling #13801S) and β-actin (1:1000) (Cell Signaling #4967S). Secondary anti-rabbit and anti-mouse IgG antibodies conjugated to horse radish peroxidase (HRP) (1:2000) (Thermo) were used and bands were identified using the ECL Plus Western Blotting Substrate (Pierce Biotechnology).

### Proliferation assays

For proliferation assays, cells were manually counted using a hemocytometer using trypan blue and 1000 cells were plated in triplicate on day 1 in DMEM supplemented with 10% fetal bovine serum (FBS) at 37 °C in a humidified atmosphere (5% CO_2_). On Day zero, 200,000 cells were counted and placed into each plate. Designated plates were detached and counted at 24-h intervals to identify cell proliferation. All experiments were repeated in triplicate. Mean cell count was compared as t-test.

### Limiting dilution assays

The BD FACS Aria II was used to sort cells in triplicate rows with corresponding serial dilutions in 96-well ultra-low attachment plates (Corning) in 200 uL of serum-free DMEM/ F12 medium, 10 ng/mL epidermal growth factor (Biosource), 20 ng/mL basic fibroblast growth factor (Invitrogen, Carlsbad, CA), 2% B-27 Supplement (Invitrogen), 10 μg/mL insulin, and 1 μg/mL hydrochloride (Sigma-Aldrich) in each well. Tumor spheres were observed at 10 days under a phase-contrasted microscope. Wells with at least one tumor sphere present were considered positive for tumor sphere initiation, and wells that failed to produce a tumor sphere were considered negative. Results were analyzed using Extreme Limited Dilution Analysis (ELDA) to calculate a corresponding stem cell frequency (http://bioinf.wehi.edu.au/software/elda/).

### RNA in-situ hybridization

Patients with stage IIIC high grade serous ovarian cancer that had undergone cytoreductive surgery at our institution and subsequently treated with platinum-based chemotherapy were identified and paraffin-embedded tissue was retrieved for RNA in-situ hybridization and analysis. This was intended as a pilot study and cases were selected to represent a range of progression-free intervals after platinum-based chemotherapy. In situ hybridization result was interpreted by a board-certified clinical pathologist at our institution. Extent of staining was graded as 1, 2, or 3 corresponding to < 25% of cells, 25–50% of cells, or > 50% of cells, respectively. De-identified clinical, pathologic, treatment, and survival data were extracted from the medical record (IRB# 13–498).

### Kaplan-Meier plotter and statistical analysis

Data analysis on high-throughput screen results was performed using the Flowjo software (Tree Star), and parametric t-test was used to compare means given Gaussian distribution of data. Kaplan-Meier Plotter (KM Plotter) for serous and endometrioid ovarian cancers (http://kmplot.com/analysis/) was used to obtain survival data based on Thy-1 mRNA expression. The KM Plotter data is comprised of The Cancer Genome Atlas (TCGA), Gene Expression Omnibus (GEO), and European Genome-phenome Archive (EGA). For KM Plotter data, the selected cutoff for all experiments was set to median Thy-1 expression. All patients with serous or endometrioid ovarian cancer accordingly were included. Quality control was enabled to include only registered unbiased arrays. For patient series (Table 1), descriptive statistics, student’s t-test, one-way ANOVA, and Wilcoxon rank-sum test were used to analyze survival data (JMP Version 14). Throughout this paper, statistical significance (*P*-value) is denoted in the Figures with “*” representing *P* < 0.05 but > 0.01, “**” representing *P* < 0.01 but > 0.001, and “***” denoting *P* < 0.001.

## Supplementary information

**Additional file 1: Figure S1.** Thy-1 is associated with poorer progression-free survival in early and late stage endometrioid ovarian cancer. High expression of Thy-1 is associated with a nonsignificant trend toward shorter progression-free survival in women with Stage I/II endometrioid ovarian cancer (A, *P* = 0.37, *n* = 28), but is associated with significantly shorter progression-free survival in women with Stage III/IV endometrioid ovarian cancer (B, *P* = 0.001, *n* = 21).

**Additional file 2: Figure S2.** Thy-1 knockdown is effective in TOV211D ovarian cancer cells. Non-targeting control (sh002) and two knockdowns were generated and validated with TOV211D ovarian cancer cells via qRT-PCR (Figure S2A) and Western Blot (Figure S2B).

## Abbreviations

AWOD: Alive Without Disease
CSC: Cancer Stem Cell
DMEM: Dulbecco’s Modified Eagle Medium
DOD: Dead of Disease
EOC: Epithelial Ovarian Cancer
FACS: Fluorescence-Activated Cell Sorting
FBS: Fetal Bovine Serum
GFP: Green Fluorescent Protein
GPI: Glycosylphosphatidylinositol
HTS: High Throughput Screen
IDS: Interval Debulking Surgery
ISH: In Situ Hybridization
KM: Kaplan-Meier
med: Median
NACT: Neoadjuvant Chemotherapy
NS: Nonsignificant
OS: Overall Survival
OSR: Overall Survival from Recurrence
PCR: Polymerase Chain Reaction
PFS: Progression-Free Survival
STR: Short Tandem Repeats
uq: Upper Quartile
