# Supplementary figures and images for: Thy-1 predicts poor prognosis and is associated with self-renewal in ovarian cancer
Source: J Ovarian Res. 2019 Nov 17;12:112. doi: 10.1186/s13048-019-0590-5 (PMC6858973; doi:10.1186/s13048-019-0590-5)

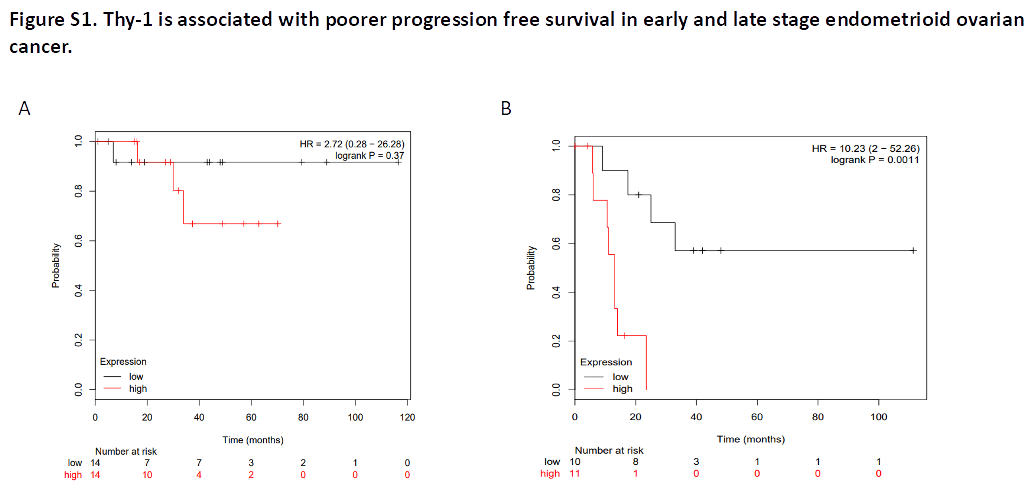

Supplement: Supplementary file 1 — Additional file 1: Figure S1. Thy-1 is associated with poorer progression-free survival in early and late stage endometrioid ovarian cancer. High expression of Thy-1 is associated with a nonsignificant trend toward shorter progression-free survival in women with Stage I/II endometrioid ovarian cancer (A, P = 0.37, n = 28), but is associated with significantly shorter progression-free survival in women with Stage III/IV endometrioid ovarian cancer (B, P = 0.001, n = 21). [file 13048_2019_590_MOESM1_ESM.docx]

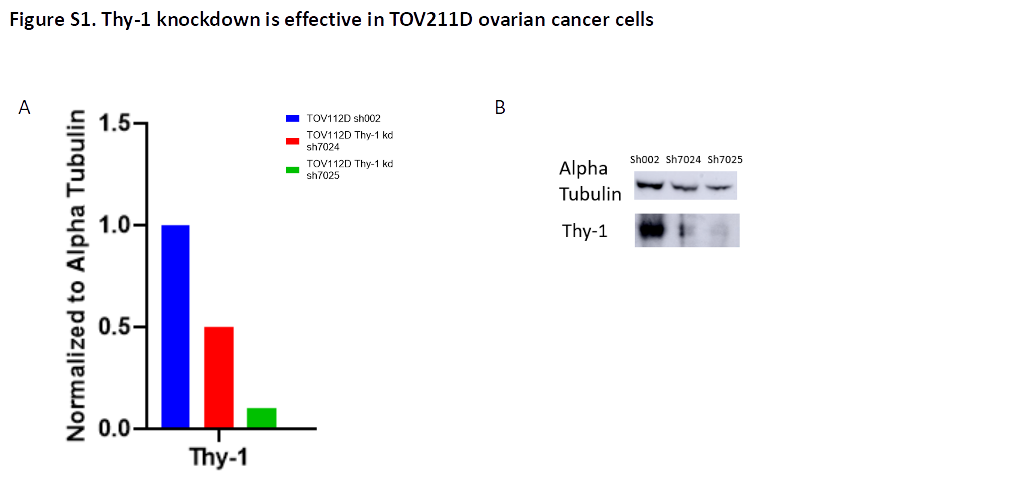

Supplement: Supplementary file 2 — Additional file 2: Figure S2. Thy-1 knockdown is effective in TOV211D ovarian cancer cells. Non-targeting control (sh002) and two knockdowns were generated and validated with TOV211D ovarian cancer cells via qRT-PCR (Figure S2A) and Western Blot (Figure S2B). [file 13048_2019_590_MOESM2_ESM.docx]
